# Practical Classification Guidelines for Diabetes in patients treated with insulin: a cross-sectional study of the accuracy of diabetes diagnosis

**DOI:** 10.3399/bjgp16X684961

**Published:** 2016-04-15

**Authors:** Suzy V Hope, Sophie Wienand-Barnett, Maggie Shepherd, Sophie M King, Charles Fox, Kamlesh Khunti, Richard A Oram, Bea A Knight, Andrew T Hattersley, Angus G Jones, Beverley M Shields

**Affiliations:** NIHR clinician scientist and StR in endocrine and diabetes, and general internal medicine;; NIHR clinician scientist and StR in endocrine and diabetes, and general internal medicine;; NIHR clinician scientist and StR in endocrine and diabetes, and general internal medicine;; NIHR clinician scientist and StR in endocrine and diabetes, and general internal medicine;; Research and Development Unit, Northampton General Hospital, Northampton.; Diabetes Research Centre, College of Medicine, Biological Sciences and Psychology, University of Leicester and the Leicester Diabetes Centre (Air Wing), Leicester General Hospital, Leicester.; NIHR clinician scientist and StR in endocrine and diabetes, and general internal medicine;; NIHR clinician scientist and StR in endocrine and diabetes, and general internal medicine;; NIHR clinician scientist and StR in endocrine and diabetes, and general internal medicine;; NIHR clinician scientist and StR in endocrine and diabetes, and general internal medicine;; University of Exeter Medical School and Royal Devon and Exeter NHS Foundation Trust, Exeter NIHR Clinical Research Facility, Barrack Road, Exeter.

**Keywords:** diabetes mellitus, C-peptide, general practice, insulin-treated diabetes, type 1/type 2 classification, type 1/type 2 diagnosis

## Abstract

**Background:**

Differentiating between type 1 and type 2 diabetes is fundamental to ensuring appropriate management of patients, but can be challenging, especially when treating with insulin. The 2010 UK Practical Classification Guidelines for Diabetes were developed to help make the differentiation.

**Aim:**

To assess diagnostic accuracy of the UK guidelines against ‘gold standard’ definitions of type 1 and type 2 diabetes based on measured C-peptide levels.

**Design and setting:**

In total, 601 adults with insulin-treated diabetes and diabetes duration ≥5 years were recruited in Devon, Northamptonshire, and Leicestershire.

**Method:**

Baseline information and home urine sample were collected. Urinary C-peptide creatinine ratio (UCPCR) measures endogenous insulin production. Gold standard type 1 diabetes was defined as continuous insulin treatment within 3 years of diagnosis and absolute insulin deficiency (UCPCR<0.2 nmol/mmol ≥5 years post-diagnosis); all others classed as having type 2 diabetes. Diagnostic performance of the clinical criteria was assessed and other criteria explored using receiver operating characteristic (ROC) curves.

**Results:**

UK guidelines correctly classified 86% of participants. Most misclassifications occurred in patients classed as having type 1 diabetes who had significant endogenous insulin levels (57 out of 601; 9%); most in those diagnosed ≥35 years and treated with insulin from diagnosis, where 37 out of 66 (56%) were misclassified. Time to insulin and age at diagnosis performed best in predicting long-term endogenous insulin production (ROC AUC = 0.904 and 0.871); BMI was a less strong predictor of diabetes type (AUC = 0.824).

**Conclusion:**

Current UK guidelines provide a pragmatic clinical approach to classification reflecting long-term endogenous insulin production; caution is needed in older patients commencing insulin from diagnosis, where misclassification rates are increased.

## INTRODUCTION

Correctly classifying patients with diabetes with type 1 or type 2 is fundamental to ensuring they receive correct management.^1^–^3^ In clinical practice this can be challenging, with 7–15% patients misclassified in England, and large variations in practice.^4^–^7^

**Figure 1.**
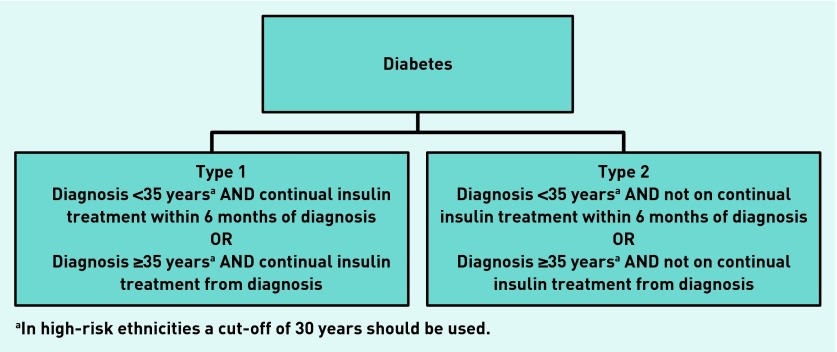
***UK Practical Classification Guidelines for Diabetes (extract showing algorithm of classification guidelines for type 1 and type 2 diabetes).^4^***

Historical lack of clear clinical guidelines for diabetes classification is likely to have contributed to this variation. International guidelines from the World Health Organization^8^ and the American Diabetes Association^9^ base classification on underlying aetiology, with type 1 described as a destruction of beta cells leading to absolute insulin deficiency. However, these guidelines do not provide clear criteria or classification pathways for clinical use.^8^,^9^ A pragmatic classification algorithm (Figure 1) was thus developed in 2010 by key diabetes stakeholders in the UK, and published by the Royal College of General Practitioners (RCGP) and (the previously existing) NHS Diabetes in their *Coding, Classification and Diagnosis of Diabetes* document.^4^ This uses age at diagnosis and time to commencing insulin treatment from diagnosis as its diagnostic criteria. The efficacy of this algorithm has not yet been tested on a large cohort of patients with diabetes.

The fundamental difference between type 1 and type 2 diabetes is the rapid development of absolute insulin deficiency in type 1, forming the basis of their different treatment and management. Patients with type 1 require accurate insulin dose replacement;^10^,^11^ patients with type 2 continue to produce substantial amounts of their own insulin, responding to non-insulin therapy, or if insulin is needed good control can be achieved with non-physiological insulin regimens.^12^,^13^ Measuring endogenous insulin secretion (using C-peptide, a component of the insulin pro-hormone secreted in equimolar amounts to insulin) in longstanding diabetes may be a useful ‘gold standard’ marker of endogenous insulin production, confirming a diagnosis of type 1 versus type 2 diabetes. Development of the spot urine test urinary C-peptide creatinine ratio (UCPCR)^14^–^17^ has enabled practical testing in a community setting. UCPCR is well-correlated with mixed meal tolerance test measures,^16^,^17^ and a UCPCR cut-off of 0.2 nmol/mmol gives a sensitivity and specificity of 100% and >95% for detecting severe insulin deficiency^16^,^17^ as defined by the gold-standard mixed meal test 90-minute C-peptide level of 200 pmol/L.^18^

**How this fits in**

Correct classification as type 1 or type 2 diabetes is fundamental to appropriate diabetes management. *The UK Practical Classification Guidelines for Diabetes* published by the Royal College of General Practitioners and (the previously existing) NHS Diabetes are pragmatically based on age at diagnosis and time from diagnosis to commencing insulin treatment. This the first study testing the UK classification guidelines in a large cohort of insulin-treated patients against a gold standard classification of diabetes subtype based on presence or absence of retained endogenous insulin secretion (measured using C-peptide) >5 years post-diagnosis. The UK classification criteria correctly classified 86% of patients, with age at diagnosis and time to insulin being the best clinical predictors of long-term endogenous insulin production.

Therefore, this study aimed to determine the reliability of the 2010 *UK Practical Classification Guidelines*^4^ to correctly classify diabetes in a large cohort of insulin-treated participants against ‘gold-standard’ classification based on measurement of C-peptide, in those with diabetes of ≥5 years’ duration. Although UCPCR can be used at any stage in diabetes to confirm endogenous insulin levels, in the current study ≥5 years’ duration was chosen to avoid misclassifying people with early type 1 who may have been still producing their own insulin.

## METHOD

### Participants

Adults with insulin-treated diabetes centred in/around three UK centres (Exeter, Northampton and Leicester) were sent letters before attending routine diabetes appointments or retinal screening (in primary care, both urban and rural, and secondary care). Those expressing an interest in participating either by returning an expression of interest form in advance, or when arriving for their routine appointment, were formally consented on the same day, and provided the research team with data on:

- age at diagnosis;
- weight at diagnosis;
- current age;
- weight and height;
- treatment;
- time to insulin from diagnosis; and
- ethnicity.

Body mass index (BMI) at diagnosis and recruitment were calculated where possible; weight at diagnosis for those diagnosed as children converted to the adult equivalent using the *UK Child Growth Reference Standards*.^19^ Participants were also given a boric acid-containing urine specimen pot and padded stamped addressed envelope. They were asked to collect a urine sample for UCPCR^14^ 2 hours after their largest meal of a day, and post the next morning (within 24 hours) for analysis in the Exeter biochemistry laboratory. UCPCR is stable in boric acid at room temperature for at least 3 days.^14^ There was no financial incentive for participating.

### Classification of diabetes

Participants were classified as having type 1 or type 2 diabetes using the UK guidelines,^4^ (Figure 1). The authors developed ‘gold-standard’ criteria: type 1 diabetes: continuous insulin treatment within the first 3 years of diagnosis and absolute insulin deficiency (UCPCR <0.2 nmol/mmol ≥5 years post-diagnosis);^16^ type 2 diabetes: UCPCR >0.2 nmol/mmol, or UCPCR <0.2 nmol/mmol but not treated with insulin for first 3 years after diagnosis.

### Statistical analysis

Proportions of patients correctly classified by the UK guidelines according to the ‘gold standard’ C-peptide-based definition were calculated, and differences in clinical characteristics between those correctly and incorrectly categorised were explored using the Mann-Whitney test.

Diagnostic performance of continuous variables (age at diagnosis, time to insulin, BMI at diagnosis and recruitment) was assessed using receiver operating characteristic (ROC) curves. Optimal cut-offs for these variables (with maximum specificity and sensitivity for discrimination) were calculated, and this study explored whether use of these optimal cut-offs led to improvements in classification over and above the RCGP algorithm using net reclassification improvement.^20^

Detailed subgroup analysis could not be carried out on the Asian patients due to small numbers. Analysis was carried out on Stata (version 13.1) and R (version 3.1.2).

## RESULTS

In total, 601 white European and 30 Asian patients who had had diabetes for ≥5 years responded. Table 1 shows the characteristics of participants per classification

**Table 1.** Participant characteristics

	**Overall**	**Gold standard type 1 diabetes**	**UK guidelines type 1 diabetes**	**Gold standard type 2 diabetes**	**UK guidelines type 2 diabetes**
Age at recruitment, median years (IQR)	64 (53–73)	54 (41–64)	53 (41–64)	68 (60–74)	68 (61–75)
Sex, % male	58.2	48.7	52.7	62.8	61.4
BMI at recruitment, median (IQR)	28.7 (25.3–33.3)	26.5 (23.1–29.3)	26.8 (23.8–29.7)	29.7 (26.6–34.5)	30 (26.6–34.1)
Age at diagnosis, median years (IQR)	45 (30–56)	24 (12–36)	25 (13–39)	50 (42–59)	50 (43–58)
BMI at diagnosis, median (IQR)	27 (23.9–32.0)	21.8 (19.8–26.3)	22.9 (20.0–27.6)	28.4 (25.4–32.9)	28.3 (25.2–33.6)
Latest HbA1c, % (IQR)	8.0 (7.3–8.8)	8.1 (7.4–8.9)	8.0 (7.3–8.9)	7.9 (7.2–8.8)	7.9 (7.3–8.8)
Insulin, IU/kg/24 hours (IQR)	0.64 (0.44–0.90)	0.61 (0.50–0.84)	0.61 (0.49–0.88)	0.65 (0.42–0.93)	0.64 (0.43–0.92)
UCPCR, nmmol/mmol, median (IQR)	0.6 (0.03–1.60)	0.019 (0.019–0.03)	0.019 (0.019–0.22)	1.19 (0.59–2.25)	1.1 (0.4–2.1)

*BMI = body mass index. IQR* = *interquartile range. UCPCR* = *urinary C-peptide creatinine ratio.*

### UK guidelines versus gold standard

The UK clinical classification criteria were compared with the gold standard C-peptide based criteria for defining type 1 and type 2 diabetes in the cohort of 601 white European patients. In total, 514 (86%, 95% confidence interval [CI] = 83 to 88) patients overall were correctly classified by the UK guidelines when compared with the gold standard criteria.

**Figure 2.**
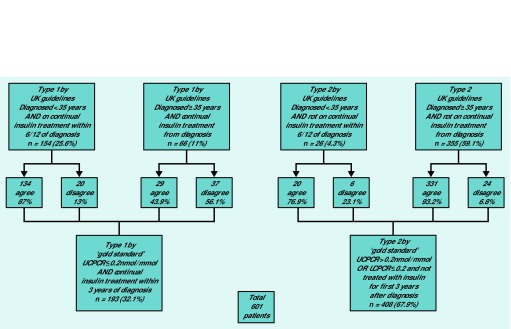
***Classification of type of diabetes according to UK guidelines’ clinical criteria compared to ‘gold standard’ C-peptide-based criteria.***

**Figure 3.**
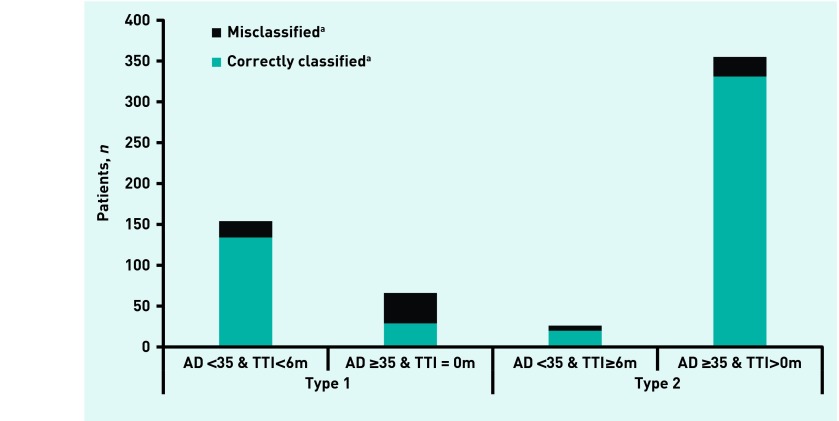
***Proportion of patients classified as having type 1 or type 2 diabetes according to the UK guidelines.^a^According to C-peptide-derived gold standard definition. AD = age at diagnosis. TTI = time to insulin from diagnosis.***

Figure 2 shows 163 out of 193 patients (84%, 95% CI = 79 to 89) were correctly classified with type 1 diabetes, and 351 out of 408 (86%, 95% CI = 82 to 89) with type 2 diabetes. The extent of the agreement between the classifications of diabetes type using the UK guidelines compared with the gold standard is evident in Figure 3.

In the Asian group, the criteria (taking note of the age cut-off of 30 years for high-risk ethnicities) performed less well, classifying only 21 out of 30 (70%) correctly (*P* = 0.02 for comparison with white Europeans) (data not shown). Three out of four (75%) were correctly classified with type 1 diabetes, and 18 out of 26 (69%) with type 2 diabetes.

### Misclassifications

Of patients misclassified by the UK guidelines’ clinical criteria in comparison with the gold standard (*n* = 87), most (*n* = 57, 66%, Figure 2) were misclassified as having type 1 diabetes and were producing substantial endogenous insulin ≥5 years post diagnosis (data not shown). Thirty out of 87 patients (34%) were misclassified as having type 2 diabetes (Figure 2); these individuals were severely insulin deficient and had started insulin treatment within 3 years of diagnosis.

The majority of misclassifications (eight out of nine) in the Asian group were also cases in which the UK guidelines’ criteria suggested type 1 diabetes (using the UK guidelines’ age cut-off of 30 years for high-risk ethnicities) but the patients were still producing their own insulin (data not shown). Most patients who were misclassified as having type 1 diabetes were diagnosed aged ≥35 years, and were given insulin immediately. According to UK guidelines, 66 patients had type 1 diabetes by these criteria, however 37 of these (56%) had a UCPCR of >0.2 nmol/mmol and so, by gold standard criteria, had type 2 diabetes (Figure 2).

Those misclassified as having type 1 diabetes were older than those correctly classified (median age 44 years [interquartile range {IQR} 30–59 years] versus 20 years [IQR 11–30 years], *P* <0.001) and had a higher BMI at diagnosis (26.4 kg/m^2^ [IQR 23–30.3 kg/m^2^] versus 21.8 kg/m^2^ [IQR 18.9–25.4kg/m^2^], *P* = 0.002) (data not shown).

In contrast, those who were insulin deficient but were incorrectly classified by UK guidelines as having type 2 diabetes commenced insulin treatment more quickly than those correctly classified as having type 2 diabetes (time to insulin from diagnosis 12 months [IQR 2–18 months]) versus 84 months [IQR 42–138 months], *P* <0.001), had lower BMI (22.5 kg/m^2^ [IQR 21.1–26.3 kg/m^2^] versus 28.1 kg/m^2^ [IQR 25.4–33.3 kg/m^2^], *P* <0.001), and were younger at diagnosis (44 years [IQR 35–56 years] versus 51 years [IQR 43–59 years], *P*= 0.014).

### Optimal clinical criteria

**Figure 4.**
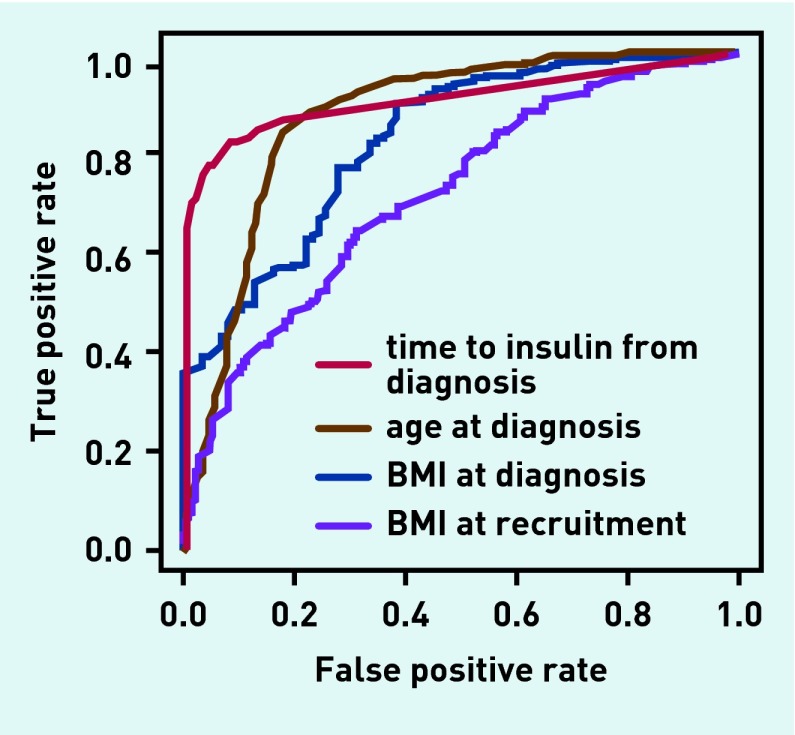
***Receiver operating characteristic curve for discriminating between type 1 and type 2 diabetes (Based on the gold standard definition).***

ROC curves were used to examine the discriminative ability of key clinical criteria: time to insulin, age at diagnosis, BMI at diagnosis, and BMI at recruitment (Figure 4). They were also used to identify the best cut-offs for classification based on the ‘gold standard’ criteria. An area under the curve (AUC) equal to 1 represents the perfect discrimination between types of diabetes, and an AUC of >0.8 is generally deemed clinically useful.

The most discriminatory individual characteristic was months from diagnosis to insulin treatment (AUC 0.904, 95% CI = 0.88 to 0.93), with the optimal cut-off at 12 months. In total, 91.5% patients were correctly classified as having type 1 diabetes and 82.1% were correctly classified as having type 2 diabetes (data not shown).

Age at diagnosis was also a useful discriminator between type 1 and type 2 diabetes (AUC 0.871, 95% CI = 0.84 to 0.90), with the optimal cut-off being ≤39 years for type 1 diabetes. This correctly classified 81.9% of patients with type 1 and 84.3% of those with type 2 diabetes (data not shown).

BMI at diagnosis gave an AUC of 0.824 (95% CI = 0.77 to 0.87; data were available in 359 of 601 [59.7%] patients only), with the optimal cut-off being ≤23.1 kg/m^2^. However, although this correctly classified 89.4% of those with type 2 diabetes, it only classified 65.7% of patients with type 1 correctly .

BMI at recruitment was even less discriminatory, with an AUC of 0.72 (95% CI = 0.67 to 0.76) and an optimal cut-off of 28.0 kg/m^2^; this correctly classified 66.8% of people with type 2 diabetes, and 61.8% of people with type 1 diabetes.

### Modifying the guidelines’ clinical criteria

The UK guidelines use age at diagnosis and time to insulin as the classification criteria to differentiate between type 1 and type 2 diabetes. On the basis of the ROC curve data, the optimal cut-offs for time to insulin (12 months), age at diagnosis (39 years), BMI at diagnosis (23.1 kg/m^2^), and recruitment (28.0 kg/m^2^) were incorporated into modified criteria in various combinations to see whether they improved diagnostic accuracy. Aiming for a sensitivity and specificity of >80% (equivalent to an ROC AUC of >0.8), none were superior to the UK guidelines as improvements in sensitivity led to greater decreases in specificity and vice versa.

The best-performing alternative was the combination of an age cut-off of 39 years and time to insulin of 12 months; this improved the correct classification of those with type 2 diabetes to 94%, but reduced to 78.3% those correctly classified with type 1 diabetes. In general, adding BMI at diagnosis or time of recruitment improved the proportion of those with type 2 diabetes that were correctly classified, but markedly reduced the proportion correctly classified with type 1 diabetes.

## DISCUSSION

### Summary

The study results show that the UK guidelines are an accurate method of predicting long-term endogenous insulin production and perform well in correctly classifying patients with insulin-treated diabetes based on the development of absolute insulin deficiency using endogenous insulin levels and time to insulin from diagnosis. This supports the guidelines’ use as a beneficial, pragmatic way of classifying patients. When all patients with diabetes are considered, the authors hypothesise that the performance of the UK guidelines will be even better because the vast majority of patients who are not treated with insulin will be correctly classified as having type 2 diabetes.

Patients diagnosed at an older age (≥35 years) in whom insulin treatment commenced at diagnosis are at the highest risk of being misclassified when using the UK guidelines.

In clinical practice, emphasis is often placed on BMI to help differentiate between type 1 and type 2 diabetes but the study findings presented here indicate that, among patients treated with insulin, time to insulin and age at diagnosis are better predictors of diabetes subtype than BMI. Median BMI at diagnosis of those with type 1 diabetes by the gold standard criteria was lower than in those with type 2 diabetes: 21.8 kg/m^2^ versus 28.1 kg/m^2^ (*P* <0.001 but the interquartile ranges overlapped (19.8– 26.3 kg/m^2^ and 25.4–32.9 kg/m^2^). By the time of recruitment (that is, ≥5 years from diagnosis), the difference in BMI between those with type 1 and type 2 diabetes was smaller (26.5 kg/m^2^ [23.1–29.3 kg/m^2^] versus 29.7 kg/m^2^ [26.6–34.5 kg/m^2^]) although still significant (*P* <0.001), and the ROC AUC was low, highlighting the reduced discriminative ability of this as a clinical marker to differentiate between type 1 and 2 diabetes once the patient was receiving insulin.

### Strengths and limitations

This study comprised patients who had had diabetes for ≥5 years. If considering all patients with diabetes, the misclassification rate of 14% is likely to be much lower: patients who are treated with tablets or diet who were diagnosed ≥5 years ago are likely to have been correctly diagnosed with type 2 diabetes. In patients with a diabetes duration of <5 years, some patients with type 1 diabetes may be still producing insulin (the ‘honeymoon’ period) and not yet treated with insulin, although it is rare for patients with type 1 diabetes to be without insulin for prolonged periods. Due to recruitment locations and difficulty in recruiting Asian patients,^21^ the majority of the recruited patients were white European; only 30 Asian patients participated. Take-up rates in the white European population were high, and participants drawn from urban and rural populations, and thus the authors consider the results in this group are likely to be fairly representative for insulin-treated patients ≥5 years from diagnosis. In comparison, the authors cannot comment on the reliability of the UK guideline criteria for populations in which the prevalence of diabetes is high; further work is needed in these groups.

Limited data on BMI were available at diagnosis, due to a combination of participants not knowing their weight at diagnosis and/or missing details in GP records in patients having been diagnosed with diabetes ≥5 years ago. Improved recording of such details in those newly diagnosed with diabetes over the last few years means the authors consider this information is likely to be more available in any future studies.

The gold standard criteria used a UCPCR cut-off of 0.2 nmol/mmol, which has a sensitivity and specificity of 100% and >95% respectively to detect absolute insulin deficiency.^16^,^17^ It is the best gold standard available in this context, being practical for use in large numbers of adults living in the community. Insulin treatment has the potential to suppress endogenous insulin,^22^–^24^ but the findings presented here show that this rarely affects diabetes classification.^24^ In addition, it should be noted that the small possibility of an overdiagnosis of type 1 diabetes is a safer direction of error than the opposite.

### Comparison with existing literature

Previous reports on the misclassification of diabetes^4^,^6^,^25^,^26^ were mainly based on contraindications in coding rather than on gold standard definitions of insulin deficiency.^18^,^27^,^28^ These reports have attempted to assess accuracy of recorded diagnosis on the basis of electronically recorded data. Although this may detect patients who are miscoded, for example as having type 1 diabetes but are not on insulin 10 years postdiagnosis, it is less likely to detect patients who are misdiagnosed, for example in receiving insulin despite high endogenous insulin levels several years after diagnosis. *The UK Practical Classification Guidelines for Diabetes*^4^ use very simple clinically available information to classify patients from scratch, and the authors have assessed their accuracy using a gold-standard diagnosis based on endogenous insulin levels and time to insulin.

A recently published systematic review identified diagnostic accuracy studies in the literature, which compared clinical criteria with C-peptide cut-offs.^7^ Age at diagnosis, time to insulin, and BMI are the clinical characteristics most frequently used to classify type 1 and type 2 diabetes, but few studies have addressed clearly which are most strongly associated with long-term C-peptide secretion.^7^ Where strength of association has been measured, time to insulin and age at diagnosis appear stronger than BMI. Again as found in the current study, combining time to insulin and age at diagnosis improved diagnostic accuracy, with BMI adding little.^7^

### Implications for research and practice

Correct classification of type 1 and type 2 diabetes is important so the appropriate treatment and management guidelines are followed;^3^,^29^ this will relate to treatment, education (for example, about dose adjustment for normal eating for those with type 1), and the monitoring of complications, all of which are based on the presence or absence of endogenous insulin.

The clinical problem facing GPs and other health professionals is that classification can be tricky at the time of diagnosis and all guidelines — including the UK classification guidelines assessed in this study — rely on information that is only available further down the line (for example, time to insulin). The gold standard classification using UCPCR ≥5 years from diagnosis, by definition, cannot completely solve this conundrum: UCPCR of >0.2 nmol/mol within 5 years of diagnosis may represent someone with type 1 diabetes who is still in the ‘honeymoon’ phase, or someone with type 2 diabetes; a UCPCR of <0.2 nmol/mmol within 5 years of diagnosis can diagnose type 1 diabetes however. Studies designed to improve classification at diagnosis, for example by using islet antibodies, are needed to address this problem.

This study has shown that the UK guidelines based on time to insulin and age at diagnosis are accurate and pragmatic for classifying patients with type 1 or type 2 diabetes. Time to insulin is subject to many influences — physician or patient factors, or guidelines for treatment in a particular area or patient population — but the high rate of correlation of diagnosis with the gold standard suggests overall timing of insulin initiation may be reasonably consistent. Clinically, where the type of diabetes is unclear, giving insulin from diagnosis is a rational decision to avoid the potential consequences of untreated type 1 diabetes, such as ketoacidosis. This study however demonstrates high rates of misclassification as type 1 diabetes in those diagnosed >35 years of age, and thus revisiting the diagnosis in these patients may be worthwhile. The authors suggest that, if there is diagnostic uncertainty, the diagnosis be reviewed, specialist advice sought, and further investigations (for example, C-peptide and islet autoantibodies) be considered.

It could be interesting to follow up those patients identified as misclassified, and those diagnosed with type 2 and still producing insulin beyond 5 years to ascertain whether some of them may be able to withdraw successfully from insulin.

The authors have concentrated on the two main types of diabetes, but recognise that there are alternative subgroups such as genetic forms of diabetes. Although rare, these are also covered by the UK guidelines and have their own criteria for diagnosis.^30^

It is important that clinicians take into account other factors that may indicate these. The term ‘latent autoimmune diabetes in adults’ (LADA) is sometimes proposed for adults with islet autoantibodies who eventually (>12 years) become severely insulin deficient, but do not require insulin for at least the first 6 months.^31^–^34^ However, LADA is not included in international guidelines for classification/treatment.

Finally, nothing was found to indicate that modification of the criteria used or the cut-offs proposed would improve their diagnostic performance. This study, like others such as that of Shields *et al,*^7^ suggest that age of diagnosis is a better clinical predictor of type 1 diabetes than BMI, which is often used clinically to determine diabetes subtype when it is not clinically obvious; this supports the fact that more emphasis should be placed on age of diagnosis in uncertain cases. This is perhaps particularly relevant in a time when the BMI of the average population is increasing.^35^,^36^

This study demonstrates that the UK Practical Classification Guidelines for Diabetes are an accurate means for differentiating between type 1 and type 2 diabetes in most instances, with time to insulin and age at diagnosis being the most discriminatory clinical characteristics. As patients aged ≥35 years who were treated with insulin from diagnosis had the highest rate of misclassification (56% classed incorrectly as having type 1 diabetes), further investigation should be considered in this subgroup.
